# Effect of the Synthetic Parameters over ZnO in the CO_2_ Photoreduction

**DOI:** 10.3390/molecules28124798

**Published:** 2023-06-16

**Authors:** Danny Zanardo, Giulia Forghieri, Elena Ghedini, Federica Menegazzo, Alessia Giordana, Giuseppina Cerrato, Elti Cattaruzza, Alessandro Di Michele, Giuseppe Cruciani, Michela Signoretto

**Affiliations:** 1 CATMAT Lab, Department of Molecular Sciences and Nanosystems, Ca’ Foscari University of Venice and INSTM-RU Ve, 30172 Venice, Italy; danny.zanardo@unive.it (D.Z.); giulia.forghieri@unive.it (G.F.); elena.ghedini@unive.it (E.G.); federica.menegazzo@unive.it (F.M.); 2 Center for Sensors and Devices, Fondazione Bruno Kessler, 38123 Trento, Italy; 3 Department of Chemistry and NIS Interdept, Centre and Consortium INSTM, University of Turin, 10125 Turin, Italy; alessia.giordana@unito.it (A.G.); giuseppina.cerrato@unito.it (G.C.); 4 Department of Molecular Sciences and Nanosystems, Ca’ Foscari University of Venice, 30172 Venice, Italy; elti.cattaruzza@unive.it; 5 Department of Physics and Geology, University of Perugia, 06123 Perugia, Italy; alessandro.dimichele@unipg.it; 6 Department of Physics and Earth Science, University of Ferrara, 44122 Ferrara, Italy; cru@unife.it

**Keywords:** zinc oxide, surface properties, opto-electronic properties, carbon dioxide photoreduction, carbon dioxide adsorber

## Abstract

Zinc oxide (ZnO) is an attractive semiconductor material for photocatalytic applications, owing to its opto-electronic properties. Its performances are, however, strongly affected by the surface and opto-electronic properties (i.e., surface composition, facets and defects), in turn related to the synthesis conditions. The knowledge on how these properties can be tuned and how they are reflected on the photocatalytic performances (activity and stability) is thus essential to achieve an active and stable material. In this work, we studied how the annealing temperature (400 °C vs. 600 °C) and the addition of a promoter (titanium dioxide, TiO_2_) can affect the physico-chemical properties of ZnO materials, in particular surface and opto-electronic ones, prepared through a wet-chemistry method. Then, we explored the application of ZnO as a photocatalyst in CO_2_ photoreduction, an appealing light-to-fuel conversion process, with the aim to understand how the above-mentioned properties can affect the photocatalytic activity and selectivity. We eventually assessed the capability of ZnO to act as both photocatalyst and CO_2_ adsorber, thus allowing the exploitation of diluted CO_2_ sources as a carbon source.

## 1. Introduction

Zinc oxide (ZnO) is an n-type semiconductor (SC) characterized by a wide direct bandgap (3.37 eV), a large excitonic biding energy (60 meV) [1] and a high electron mobility (115−205 cm^2^·V^−1^·s^−1^) [2]. Such opto-electronic properties make ZnO suitable in the fabrication of electronic and opto-electronic devices such as light-emitting diodes (LED) [3], thin film transistors (TFT) [4] and solar cells [5]. Owing to these properties, ZnO is also an attractive material in photocatalysis and it has been studied for wastewater photo-remediation [6], hydrogen production [7], and carbon dioxide (CO_2_) photoreduction [8]. The latter is a particularly appealing application because it enables the conversion of an abundant waste and greenhouse gas, CO_2_, into useful fuels or chemicals by operating in mild reaction conditions (T < 100 °C) [9,10].

The synthesis of ZnO-based photocatalysts generally involves solvothermal [11,12] or hydrothermal [11,12,13] techniques, which, however, require harsh conditions. A milder alternative is a wet-chemistry-based route such as precipitation, though this has been reported less as a synthetic strategy for photocatalysts [14,15]. Regardless of the preparation method, the synthetic parameters strongly affect the material’s physical–chemical properties, in turn reflected in the photocatalytic performances. For example, Zhang et al. observed the use of an increasing annealing temperature of a zinc precursor to decrease the surface defects, which are supposed to act as trap and active sites, thus decreasing the overall activity of ZnO [16]. Liu et al. noticed the annealing temperature improved activity by reducing bulk defects responsible for recombination phenomena but then decreased above 500 °C owing to the removal of surface oxygen vacancies (V_O_), supposed to be the active sites [17]. The photocatalytic performances can be also tuned by the modification of the ZnO surface. In this regard, Li et al. observed an improved photo-stability of ZnO via the addition of TiO_2_, supposed to stabilize the material against light-induced degradation (photo-corrosion) and thus limiting the activity loss over time [14]. Surface functionalization has also been reported to be useful in reducing the charge carrier recombination phenomenon owing to heterojunction formation [18] and to boost the surface charge transfer by decreasing the kinetic energy barrier (co-catalysis) [19]. In summary, understanding how the synthetic route affects both the material properties and catalytic performances is essential to achieving an active and stable photocatalyst.

The basicity of ZnO is another interesting property of this material due to its interaction with an acid gas such as CO_2_ [20]. Indeed, weakly basic (or non-basic) semiconductors such as titanium dioxide (TiO_2_) have a poor surface affinity with CO_2_ in the presence of H_2_O [21]. This makes a high CO_2_/H_2_O ratio necessary to favor CO_2_ adsorption, improving its conversion and depressing the hydrogen evolution reaction (HER), a side process competing with CO_2_ reduction [22]. Nevertheless, the requirement of a CO_2_-rich stream for this purpose does not match the abundant diluted sources of this gas, requiring additional energy-consuming steps to enrich the stream [23]. This issue has been overcome by using composite materials of the photocatalyst blended with a non-photoactive CO_2_ adsorber, enabling the composite to both capture CO_2_ and convert it upon irradiation [24,25]. ZnO being active toward both CO_2_ photoreduction [11] and adsorption [20], it is an appealing alternative to the above-mentioned composites, allowing its direct utilization for this purpose. However, to date, no applications of ZnO as a hybrid CO_2_ capture–photoconverting material have been reported yet.

Through this work, some photocatalytic ZnO materials were prepared via a wet-chemistry route. They were then characterized, aiming to understand how the synthetic parameters (annealing temperature and presence of a TiO_2_ promoter) can affect their physico-chemical properties. The materials were tested in the gas-phase CO_2_ photoreduction with water, aiming to correlate their properties to this activity as well. Finally, ZnO was assessed in hybrid CO_2_ capture–photoconversion.

## 2. Result and Discussion

### 2.1. Structural and Morphological Characterization

The first step of the synthetic method used in this work (wet-base precipitation) affords a phase-pure zinc hydroxy carbonate (Zn_5_(OH)_6_(CO_3_)_2_, Reaction 1), as evidenced by the X-ray diffraction (XRD) pattern (Figure S1b, hydrozincite, ICDD PDF card no. 72-1100). In the second step, the hydroxycarbonate undergoes an endothermic decomposition peaking at ca. 265 °C, releasing CO_2_ and H_2_O gases, as shown by the thermal gravimetry–differential thermal analysis (TG-DTA, Figure S2b), and affording the desired zinc oxide material (ZnO, Reaction 2). Two different temperature are then chosen (400 °C and 600 °C) for the annealing of zinc hydroxy carbonate, and a TiO_2_ promoter is added in two different ways, as described in Section 3.2.

Reaction 1. 5ZnSO_4_(aq) + 5Na_2_CO_3_(aq) + 3 H_2_O (l) → Zn_5_(OH)_6_(CO_3_)_2_(s) + 5Na_2_SO_4_(aq) + 3CO_2_(g)

Reaction 2. Zn_5_(OH)_6_(CO_3_)_2_(s) → 5ZnO(s) + 2CO_2_(g) + 3H_2_O(g)

**Figure 1:**
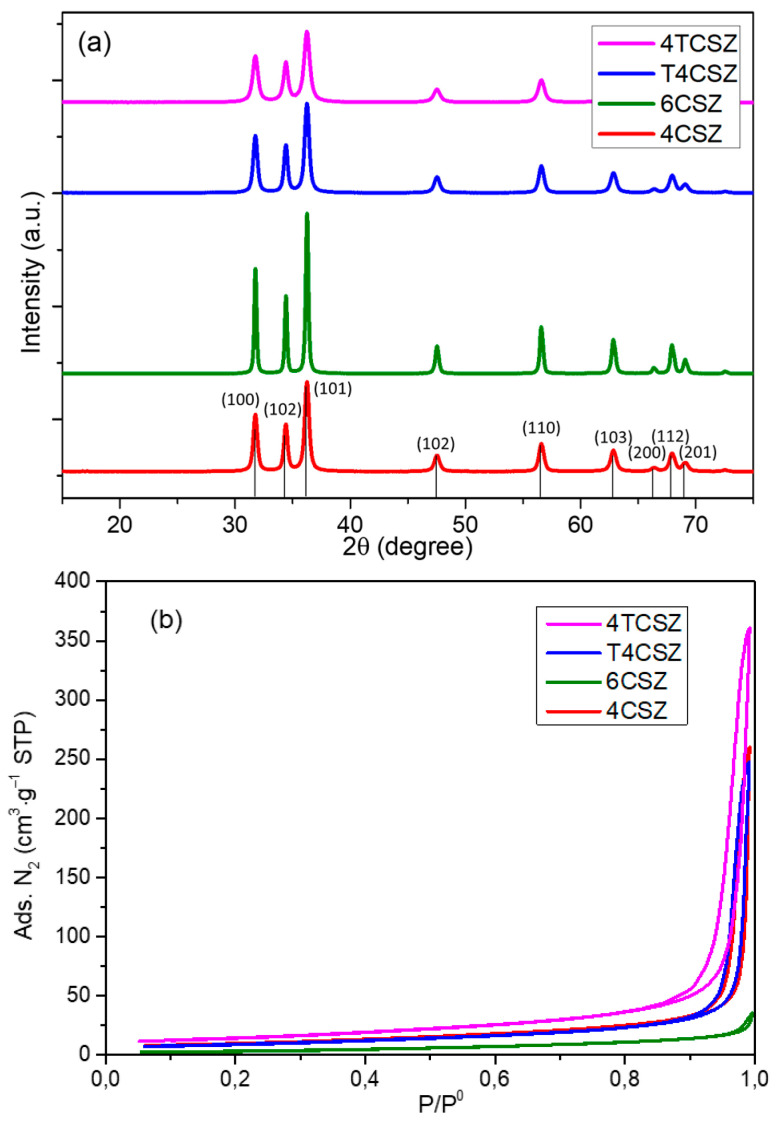
(**a**) XRD diffractograms and (**b**) N_2_ physisorption isotherms of ZnO materials.

The XRD pattern of the synthetized materials (Figure 1a) shows a crystalline and phase-pure ZnO (zincite, ICDD PDF card no. 36-1451). The presence of zincite is further confirmed by the vibrational E_2_ low (99 cm^−1^) and E_2_ high (437 cm^−1^) modes [26] evident in the Raman spectra (Figure S3). It is worth mentioning that this phase purity can be achieved by a proper selection of the precipitating agent (Na_2_CO_3_ in this study); otherwise, mixed phases with poor photoactivity could be obtained, such as by using NaOH [15]. No peaks ascribable to TiO_2_ phases were observed on Ti-containing samples (T4CSZ and 4TCSZ) in XRD and Raman data, suggesting the presence of TiO_2_ in the form of tiny NPs, amorphous material or incorporated in the ZnO lattice [27]. The synthetic parameters are also observed to affect the primary particle (crystallite) size, increasing in the order 4TCSZ < 4CSZ ≈ T4CSZ < 6CSZ (Table 1). The higher annealing temperature (6CSZ) and the TiO_2_ addition before ZnO annealing (4TCSZ) can effectively tune this property, while the post-annealing TiO_2_ promotion (T4CSZ) has negligible effects compared to the unpromoted sample annealed at lower temperature (4CSZ).

The gas N_2_ physisorption analyses (Figure 1b) show isotherms ascribable to macro-mesoporous materials for all the examined samples [28]. The specific surface area (SSA, Table 1) increases in the order 6CSZ < 4CSZ ≈ T4CSZ < 4TCSZ, which is in good agreement with the trend observed by the XRD (smaller crystallites afford higher SSA).

**Figure 2:**
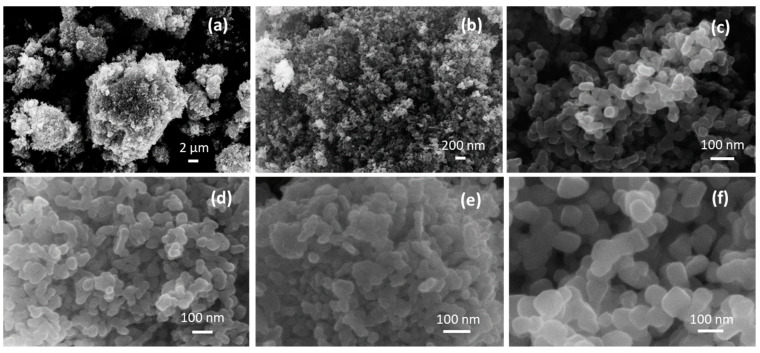
(**a**–**c**) SEM images of 4CSZ at different magnifications and SEM images of (**d**) T4CSZ, (**e**) 4TCSZ, (**f**) 6CSZ.

The scanning electron microscopy (SEM) images show the presence of micrometer-sized agglomerates (Figure 2a), in turn composed of primary NPs joined together (Figure 2b,c). The lower annealing temperature in 4CSZ, T4CSZ and 4TCSZ afforded small globular particles (Figure 2c–e). Furthermore, on 4TCSZ, the particles seem to be more tightly packed, while the higher annealing temperature of 6CSZ (Figure 2f) leads to an enlargement of the particles. The EDS analyses observed ca. 0.5 at. % Ti on both T4CSZ and 4TCSZ, half of the theoretical loaded amount, and it is observed to be highly dispersed on these materials, as observable by the elemental mapping (Figures S4 and S5, respectively).

**Figure 3:**
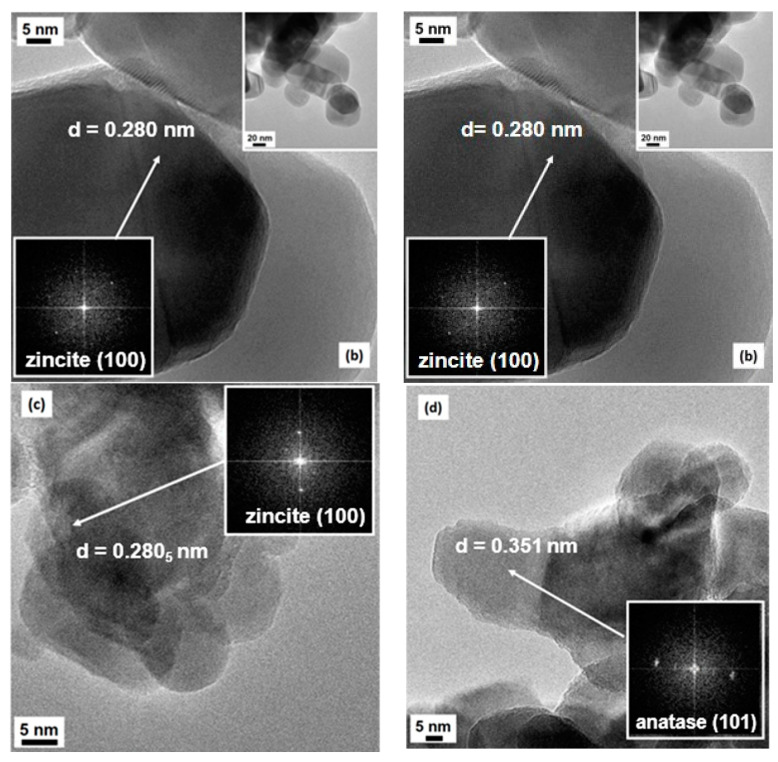
HR-TEM images of (**a**) 4CSZ, (**b**) 6CSZ, (**c**) T4CSZ and (**d**) 4TCSZ. Insets to Figure 3a,b refer to low-magnification situations, whereas the black images with bright spots in all images refer to FFT elaboration, i.e., virtual electron diffraction patterns, for all samples.

The transmission electron microscopy (TEM) analysis (Figure 3) essentially confirms the shape and the size of the particles evidenced by SEM imaging. In all cases, clear contours and high crystallinity of the particles are evinced, confirming the indications from XRD analysis: fringe patterns are often observed, clearly visible in the high-magnification images of both 4CSZ and 6CSZ, mainly at its best for the high-temperature-treated system, and supported by the FFT elaborations of the images (see Figure 3a,b and insets therein). The most frequently evidenced fringe patterns are referred to either the (101) or (100) family planes belonging to zincite (ICDD PDF card no. 36-1451): the relevant spacings are reported in Figure 3a,b, respectively, being 0.247 and 0.280 nm for the quoted crystal planes. In no cases are the typical rod-like hexagonal prism shape of ZnO crystallites observed, whereas, in some cases, hexagonal platelets can be observed. The dimensions of the crystallites were in agreement with the results from the Rietveld analysis. When TiO_2_ is added, despite the procedure of it addition, there is an even lesser variation in the typical rod-like shape of ZnO with a parallel development of a more defective structure of the outer surface of the crystallites; in fact, as seen in Figure 3c,d, less defined contours and more edged surfaces are observable, even though a high degree of crystallinity is retained, due to either ZnO (again, the (100) family planes of zincite have been singled out for the support, see Figure 3c) or the added TiO_2_. The presence of TiO_2_ has been confirmed by EDS analysis. Moreover, for the T4CSZ sample, it was possible to investigate in detail the fringe patterns present in many images (see also the FFT-simulated electron diffraction patterns): the main frequently exposed crystal planes are those of anatase, with distances of ca. 0.35 nm due to the (101) planes (ICDD PDF card no. 21-1272).

In conclusion, the size and surface morphology of the ZnO materials is affected by both the annealing temperature of the zinc hydroxy carbonate precursor and the addition of a Ti-based promoter. The annealing temperature mainly affects the size of the ZnO particles, with no impact on shape or surface morphology. The addition of TiO_2_ promoter affords the surface morphology, inducing a more “rough” surface (rich in edges). Moreover, if the TiO_2_ precursor is added after ZnO annealing, the particle size remains almost unaltered, but crystalline anatase (TiO_2_) can be detected on their surface. On the other hand, the addition of TiO_2_ on the zinc hydro-carbonate before the annealing, leads to slightly smaller ZnO particles and the absence of a TiO_2_ crystalline form, suggesting an inhibition effect on the growth of ZnO while preserving the amorphous nature of TiO2.

### 2.2. Surface and Optical Characterization

**Figure 4:**
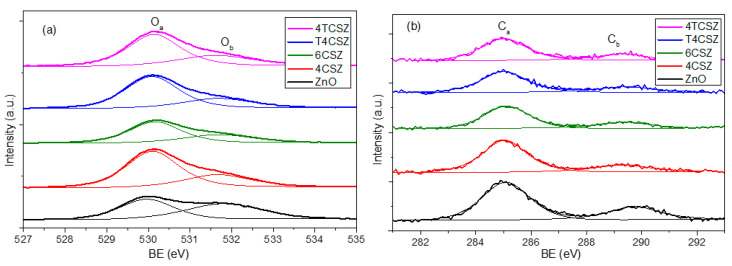
(**a**) O1s and (**b**) C1s XPS signals of ZnO materials.

The analysis of surface composition (up to ca. 10 nm depth) was performed by XPS. The structure of the XPS survey spectrum is the same for all the investigated samples: in Figure S6, a representative survey is reported. XPS reveals mainly the presence of oxygen (O) and zinc (Zn), the latter being Zn^2+^ ions, as confirmed by the Auger parameter (2010.0 eV). In all the samples, carbon (C) was detected too (concentrations below 10% at.), while in T4CSZ and 4TCSZ samples, titanium (Ti) (concentration below 1% at.) and traces of nitrogen (N) (concentration of few 0.1% at.) were also detected. The shape of the Zn2p band (not reported) is exactly the same in all samples (standard for pure ZnO too), as expected for ZnO-based compounds: this is the reason for which we used its 2p_3/2_ spin–orbit component as an internal standard to correct BE values for the surface charging, as reported in Section 3.3. The O1s region (Figure 4a) shows two types of signals: one at 530.0 eV (O_a_), ascribable to lattice O^2−^ ions [29], and another at 531.7 eV (O_b_), imputable to the hydroxyl (OH) [30] moiety or O^2−^ ions in a defective matrix (i.e., oxygen vacancy) [31]. The C1s region (Figure 4b) reveals its main signal falling at 285.0 eV (C_a_), ascribable to adventitious carbon, and another one at 289.5 eV (C_b_) related to carbonate (CO_3_^2−^) or hydroxy carbonate (HCO_3_^−^) species [32]. The Ti2p doublet (Figure S6) shows a 2p_3/2_ component centered at 458.4 eV, pointing out the presence of Ti^4+^ [33].

As the O_a_/O_b_ ratios (Table 2) are quite similar, the concentration of defect-related and/or hydroxyl species (O_b_) is supposed to be almost comparable for all the samples, with only a slightly O_b_-poor surface on 4TCSZ. The absolute concentration of surface carbonate/hydroxy carbonate (C_b_) species is found to be relatively constant as well. The most relevant difference showed by XPS analysis is the surface stoichiometry expressed as the O/Zn ratio (Table 2). Indeed, the Zn/O < 0.8 observed in samples annealed at a lower temperature (400 °C) can be ascribed to an O-poor surface, while the Zn/O > 0.8 detected on the sample annealed at a higher temperature (6CSZ) is related to a surface slightly richer in oxygen. The annealing temperature thus affects the surface stoichiometry, while the addition of TiO_2_ has basically no effects. For comparison, a pure and crystalline ZnO reference sample was analyzed, too, showing a stoichiometric surface composition (O/Zn = 1.00).

**Figure 5:**
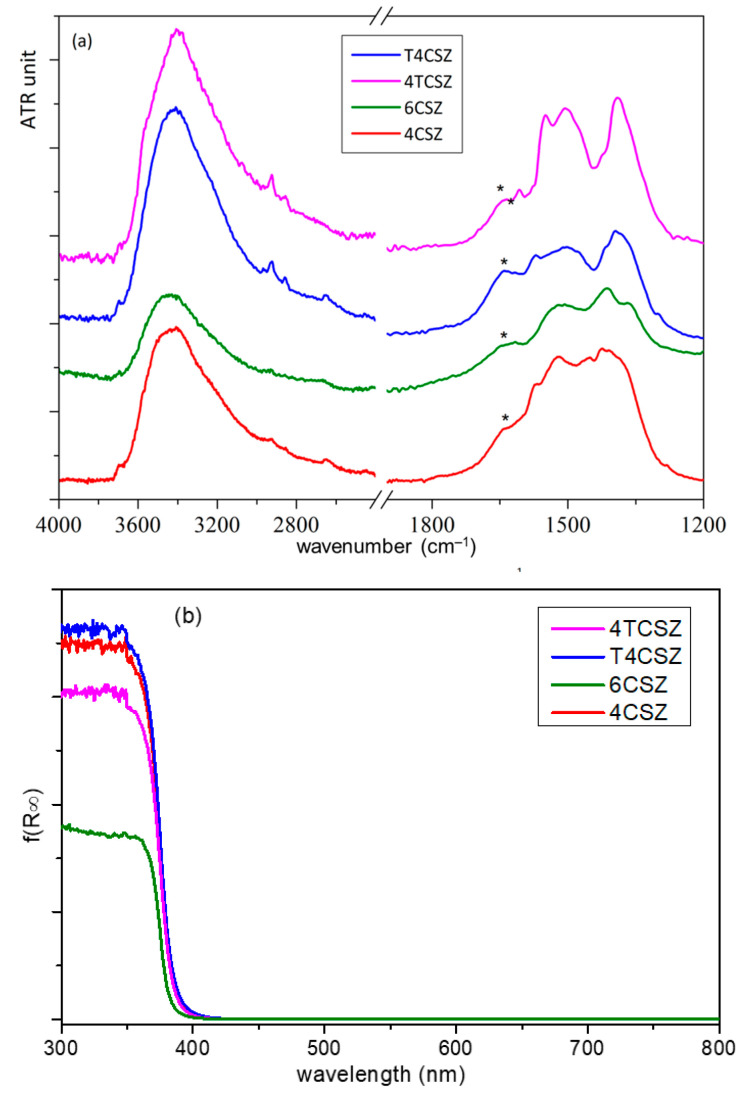
(**a**) ATR-FTIR spectra and (**b**) UV-vis absorption spectra (Kubelka–Munk function) of ZnO materials. The asterisk on ATR-FTIR spectra point out the δ_O−H_ shoulder.

The Fourier transform IR-attenuated total reflectance (FTIR-ATR) spectra of all the samples are dominated by a strong signal below 600 cm^−1^, attributable to Zn−O modes, but two other broad bands are also observable (Figure 5). The more intense band, at a high wavenumber (3750–2800 cm^−1^), is ascribable to the stretching modes of O−H groups interacting via H bonding, related to the presence of water molecules and/or surface OH groups. The other band (in 1700-1250 cm^−1^ spectral range) is composed of overlapping signals: the bending mode of OH groups (at 1640 cm^−1^, asterisk in Figure 5a) and the stretching modes of surface carbonate and bicarbonate species. Through semiquantitative analysis (Figure S8), obtained by comparing normalized spectra of the samples, both signals resulted in being less intense for 6CSZ with respect to 4CSZ, as to be expected due to the former’s higher annealing temperature. The addition of titania increases the amount of water but seems not to influence the (bi)carbonate coverage when Ti is added prior to annealing; on the contrary, the amount of (bi)carbonate decreases on T4CSZ with respect to 4CSZ. The different surface coverage can be related to the presence of crystalline titania on the surface of T4CSZ, which is absent in 4TCSZ, considering that (bi)carbonate formation is favored on the basic surface of ZnO [20], while the more acidic and oxyphilic TiO_2_ interacts preferably with water [21].

The optical absorbance (Figure 5b) shows the typical UV absorption edge of ZnO, with no appreciable features on the visible range. The band-gap (E_g_) values and the Urbach energies (E_U_), the latter ascribable to the density of intra-band defect states [34], are almost comparable between all samples (Table S1). This evidenced both the absence of a quantum confinement effect, which may arise from the diverse crystallite size [34], and a comparable defect density on all the samples, as previously supposed by XPS analysis.

### 2.3. Photoluminescence Characterization

**Figure 6:**
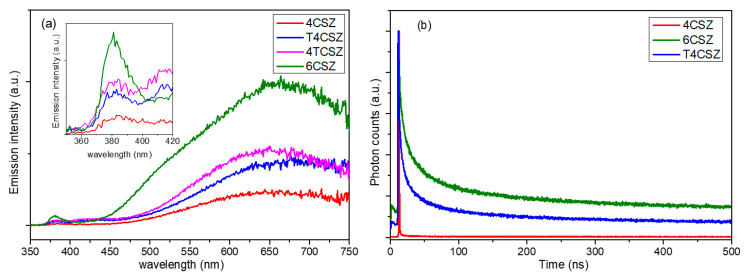
(**a**) Steady-state PL spectra and (**b**) TR-PL graph of the visible emissions (λ_EM_ = 641 nm) of ZnO-based samples.

The steady-state photoluminescence (PL) spectra (Figure 6a) show two types of emission bands, a weaker one in the UV region (inset in Figure 6a), ascribable to band-to-band radiative relaxation of the crystalline core of ZnO NPs, and a stronger one in the visible region (λ > 400 nm), related to the high surface density of native point defects [35]. Analyzing the visible emission bands in more detail, four types of features can be observed (Figure S10 and Table S2): the orange (λ_MAX_ ca. 600 nm) and red (λ_MAX_ ca. 700 nm) bands that are very intense and found in all samples, a green band (λ_MAX_ = 520 nm) that appears on 6CSZ and a weak violet band (λ_MAX_ = 420 nm) that appears in TiO_2_-containing samples (T4CSZ and 4TCSZ). The concentration of surface defects, as previously discussed, is thought to be comparable on all materials, while the visible emission intensity varies from sample to sample and increases in the order 4CSZ < T4CSZ ≈ 4TCSZ < 6CSZ. It can thus be hypothesized that some defects are involved in non-radiative decay process and others in radiative decay phenomena, in turn depending on the synthetic method of the material.

The time-resolved photoluminescence (TR-PL) decay can give some more insights into the photo-excited charge carriers’ separation and recombination phenomena, and for this purpose, we selected three representative samples: 4CSZ, 6CSZ (high-temperature annealing) and T4CSZ (TiO_2_ promotion). As shown in Figure 6b, 4CSZ exhibited a faster decay compared to T4CSZ and 6CSZ, the latter having the more persistent emission. The three lifetime parameters obtained through a tri-exponential fit of decay profiles (Table 3) can be ascribed to non-radiative recombination (τ_1_), radiative recombination (τ_2_) and energy-transfer phenomena (τ_3_). In particular, the τ_2_ parameter is related to the charge carrier’s separation efficiency [12].

The outcomes of both the PL and TR-PL suggest that the surface composition has a great impact on the charge carrier separation and recombination. The defects favored in O-rich surfaces, afforded by high-temperature annealing in 6CSZ, are supposed to improve the charge carrier’s separation efficiency and radiative recombination. On the other hand, the defects favored in Zn-rich surfaces of unpromoted 4CSZ can probably afford a faster non-radiative recombination phenomenon. However, the addition of TiO_2_ promoter can mitigate this effect, likely by the passivation of these detrimental defects [36]. Even though TiO_2_ could act as charge carrier sink, as discussed below, this mechanism is supposed to be marginal due to the weakness of the TiO_2_-related violet emission band compared to the others (Table S2).

**Figure 7:**
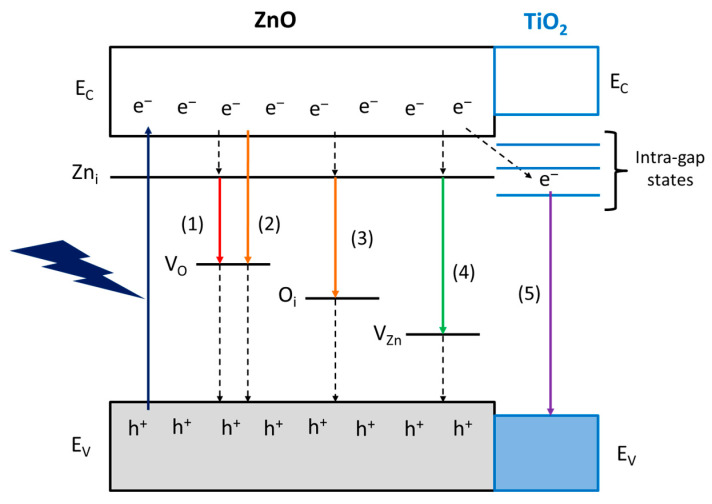
Proposed band diagram and visible emission mechanisms of ZnO materials.

Notwithstanding that there is still a large debate on the interpretation of the nature of the luminescence centers in ZnO, we propose a band diagram of the ZnO materials studied in this work (Figure 7). It is generally accepted the n-type nature of ZnO semiconductors arise from interstitial zinc (Zn_i_) defects, the energy levels of which are located 0.2-0.4 eV below the conduction band (E_C_) [37]. The red and orange emissions are supposed to be related to oxygen vacancy states (V_O_) (pathway 1 and 2, respectively) [38] or, in the O-richer surface of 6CSZ, even to interstitial oxygen (O_i_) (pathway 3) [39]. The appearance of a green emission in such sample is supposed to arise from zinc vacancies (V_Zn_) (pathway 4) [38], as they are favored on a O-richer layer.

Finally, the violet emission on TiO_2_-promoted samples could involve the transfer of photoexcited electrons to intra-gap states of TiO_2_, which act as charge carrier sinks, and eventually the radiative relaxation to the valence band (E_V_) of TiO_2_ (pathway 5). This intra-gap states mechanism is attributed to the unfavorable position of TiO_2_ E_C_ for an effective electron transfer, lying at higher energy than ZnO [40], and the intra-gap states reported for small TiO_2_ NPs, responsible for the blue-violet emission [41]. Moreover, the formation of intra-gap states in ZnO due to cation substitution by Ti^4+^, if present, is ruled out, as these states are reported to lie above the E_C_ of ZnO and not contributing to the visible emissions [42].

### 2.4. CO_2_ Photoreduction with H_2_O and Hybrid CO_2_ Capture–Photoconversion

**Figure 8:**
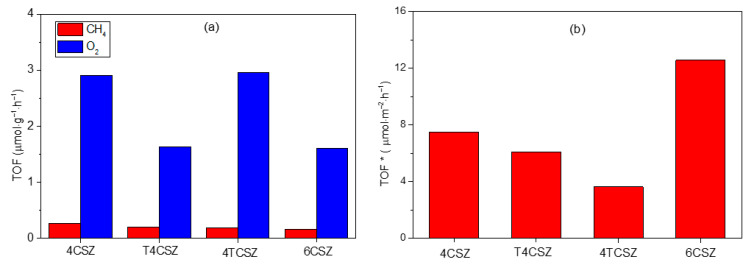
(**a**) TOF of CH_4_ and O_2_ and (**b**) TOF of CH_4_ normalized by the SSA (TOF*) of ZnO samples.

The CO_2_ photoreduction reaction is carried out using synthetized ZnO materials as photocatalysts. The only detectable products using the experimental conditions used in this work are methane (CH_4_) and oxygen (O_2_). As reported in Figure 8a, the activity toward CH_4_ production is similar in all samples (ca. 0.2 μmol∙g^−1^∙h^−1^), while it differs for O_2_ evolution, being almost doubled on 4CSZ and 4TCSZ compared to T4CSZ and 6CSZ. The detected O_2_ can be ascribed to both the water oxidation half-reaction [43] and the photo-corrosion of the ZnO [14]. The O_2_/CH_4_ ratio could give some more insights concerning the competition between these two reactions. All the samples (Figure S11, black trace) afford a ratio larger than two (stoichiometric reaction), suggesting photo-corrosion to actually be a competitive process, especially on 4TCSZ, which exhibits the largest O_2_/CH_4_ value. In T4CSZ, the slightly lower ratio compared to 4CSZ, may suggest a weak decrease in photo-corrosion.

The synthetized samples are characterized by a diverse specific surface area (SSA), which can affect the observed photo-activity. To rule out this effect, the CH_4_ and O_2_ evolution activities have been normalized per unit of SSA, affording the intrinsic activity of the samples’ surfaces (TOF*). The intrinsic activity of the surfaces towards CH_4_ formation is observed to vary as follows: 6CSZ > 4CSZ ≈ T4CSZ > 4TCSZ (Figure 8b). The O_2_ evolution, shows a slightly different trend, namely, 6CSZ > 4CSZ > T4CSZ ≈ 4TCSZ (Figure S11, blue trace), which should also be interpretated considering the O_2_/CH_4_ ratio (Figure S11, black trace). From this perspective, the material with the most active surface for both CO_2_ photoreduction and ZnO photo-corrosion is 6CSZ, while exhibiting a O_2_/CH_4_ similar to the other sample. The TiO_2_-promotion has a slightly positive effect on T4CSZ (decreased photo-corrosion), while on 4TCSZ, it decreases the surface intrinsic photo-activity and makes the CO_2_ photoreduction a less competitive process (highest O_2_/CH_4_ ratio).

The most active surface, achieved by high-temperature annealing of ZnO (6CSZ), can be related to the improved charge carriers separation efficiency and their consequent availability for the reaction and photo-corrosion (interfacial charge transfer) [12]. The effect of TiO_2_ addition strongly depends on the employed synthetic strategy. The post-annealing addition (T4CSZ), despite increasing the charge carriers separation efficiency, does not exhibit any improvement in activity compared to the TiO_2_-free material (4CSZ). This mismatch could be explained by considering the partial surface coverage of TiO_2_ NPs, on which CO_2_ is less efficiently adsorbed. Furthermore, according to the proposed band diagram (Figure 7), the electrons trapped on TiO_2_ are not able to reduce the CO_2_, which is preferentially adsorbed on the ZnO surface instead. The slightly decreased sensitiveness to photo-corrosion compared to 4CSZ can be ascribed to the partial passivation of surface sites sensitive to this phenomenon [14]. The detrimental effect of pre-annealing TiO_2_ addition on both surface activity toward CH_4_ formation and its competition over photo-corrosion could be ascribed to the slightly lower amount of O_b_ species on the surface (hydroxyl group and/or oxygen vacancy), as detected by XPS. This may result in a decreased availability of adsorption sites for the reagents, thereby increasing the competition of photo-corrosion. As the τ_2_ lifetime parameter has not been reported in this study, a less efficient charge carrier separation on 4TCSZ may not be totally excluded as well.

To elucidate the origin of the detected CH_4_, the ZnO photo-catalytic materials were assessed in both CO_2_-rich and CO_2_-free reaction media. No remarkable differences could be observed (Figure S12), and this could be partially ascribed to inorganic (bi)carbonates naturally absorbed on the surface, which are converted regardless of the presence or not of gaseous CO_2_ during the reaction, as we already observed for a TiO_2_/SiO_2_ composite material [25]. Indeed, as proven by both XPS and FTIR, inorganic (bi)carbonates are found in the synthetized materials, and they are known to be the first intermediate in CO_2_ photoreduction on ZnO materials [11]. The carbonates used in the synthesis as precipitating agents are ruled out, as the lower annealing temperature we employed (400 °C) is higher than the temperature (265 °C, Figure S2b) of carbonate decomposition of the unannealed zinc precursor (CSZ). Thus, the carbonates from the precursor are likely to be completed removed after the annealing.

**Figure 9:**
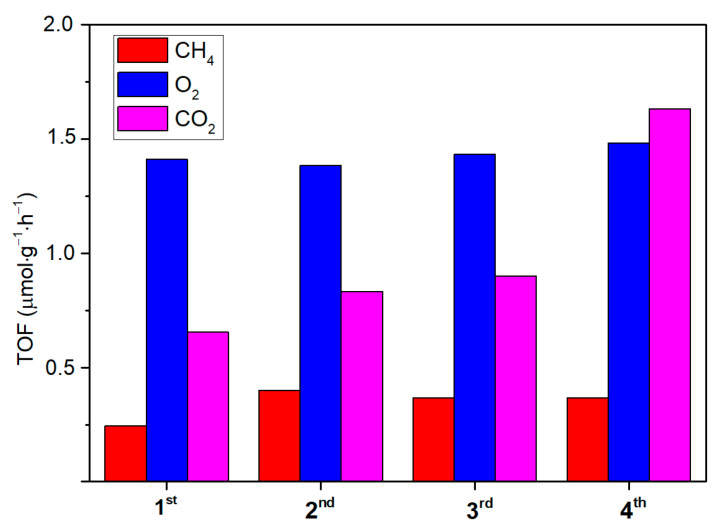
Reaction tests and re-cycles of the T4CSZ catalyst with a CO_2_-free reaction medium. The used catalyst was exposed to a dark CO_2_ flow prior to the 2nd, 3rd and 4th runs.

Owing to that, the ability of the synthetized ZnO to act as both a CO_2_ adsorber and a photoconverter is assessed on a selected active and photo-stable sample (T4CSZ). The results (Figure 9) show ZnO to be effectively able to capture CO_2_ from dark flow and then to reduce it upon irradiation in a CO_2_-free reaction medium. The CH_4_ and O_2_ yields are observed to be relatively stable, proving this material to also possess a good recyclability (CH_4_) and photo-stability (O_2_). Finally, CO_2_ is also detected after each reaction run, suggesting that the material is unable to fully convert inorganic carbonates on its surface but confirms its ability to absorb gaseous CO_2_ from diluted sources.

## 3. Materials and Methods

### 3.1. Materials

The following reagents were used as-received: ZnSO_4_∙7H_2_O (assay >99%, CAS No. 7446-20-0, Sigma Aldrich, Burlington, MA, USA), titanium (IV) isopropoxide (assay >97%, CAS No. 546-68-9, Sigma Aldrich), Na_2_CO_3_ (assay >99.5%, CAS No. 497-19-8, Sigma Aldrich), isopropanol (assay >99.7%, CAS No. 67-63-0, Sigma Aldrich) and ethanol (assay >99.9%, CAS No. 64-17-5, VWR, Radnor, PA, USA).

### 3.2. ZnO-Based Materials Synthesis

The samples were synthetized according to a previously reported wet-chemistry procedure [44]. Briefly, a 1.1 M ZnSO_4_∙7H_2_O aqueous solution was dropped into 200 mL of deionized water, keeping a constant pH 9 by using aqueous Na_2_CO_3_ 1 M. The as-obtained suspension was aged at 60 °C for 20 h, filtered, washed with deionized water to remove dissolved ions and air dried at 110 °C for 18 h. The obtained zinc-based precipitate (labelled as CSZ) was then annealed in air flow (30 mL·min^−1^) at 400 °C or 600 °C for 4 h. A 1.0 at. % TiO_2_ promoter was added by wetness impregnation of titanium (IV) isopropoxide ethanolic solution, either on unannealed Zn hydroxy carbonate or annealed ZnO, then air dried at 110 °C for 18 h and eventually annealed at 400 °C in air flow (30 mL·min^−1^) for 1 or 4 h to purposely study the effect of TiO_2_ independently from the temperature. The samples were labelled in Table 4. The samples were labelled in Table 4. The letters “T”, “C”, “S” and “Z” on the labels stand for the used precursors, “titanium”, “carbonate”, “sulphate” and “zinc”, respectively. The numbers stand for the annealing temperature.

### 3.3. Characterization

X-ray diffraction (XRD) patterns were collected on a Bruker D8 Advance DaVinci powder diffractometer using a sealed X-ray tube (copper anode; operating conditions, 40 kV and 40 mA) and a linear array detector (LynxEye), set to discriminate the Cu Kα radiation, coupled with a Ni filter to completely remove the Cu Kβ component. Data scans were performed in the 2θ range 5–90° with a 0.02° step size and point-detector equivalent counting times of 5 s/step. Quantitative phase analysis and crystallite size determination were performed using the Rietveld method, as implemented in the TOPAS v.5 program (Bruker AXS) using the fundamental parameters approach for line-profile fitting. The determination of the crystallite size was accomplished by the Double-Voigt approach and calculated as volume-weighted mean column heights based on integral breadths of peaks. N_2_ physisorption analyses were performed using a Micromeritics TriStar II Plus analyzer, recording the adsorption–desorption isotherms at –196 °C. All samples were previously outgassed at 200 °C for 2 h. The specific surface area (SSA) was evaluated using the standard BET equation [45], and comparable values were attained by performing single point SSA measurements (suitable for microporous materials). The morphology and composition were examined using Field Emission Electron Scanning Microscopy (FE-SEM) LEO 1525 ZEISS. Elemental composition and chemical mapping were determined using a Bruker Quantax EDS. The samples were deposited on adhesive carbon tape and metallized with chromium. HR-TEM images were obtained by means of a JEOL JEM 3010 transmission electron microscope, operating at 300 kV and equipped with LaB_6_ filament. Samples were dry dispersed onto Cu grids coated with amorphous carbon without any further treatment.

Reflectance UV-vis measurements were carried out on pure samples with a Cary 5000 UV-Vis spectrophotometer, plotting the spectra through the Kubelka–Munk function [46], where $R_{\infty }$ is the reflectance of an infinitely thick layer:

$$f\left(R_{\infty }\right)=\frac{{\left(1-R_{\infty }\right)}^{2}}{2R_{\infty }}$$

The bandgap (E_g_) was determined through the Tauc relation [47], plotting [$f\left(R_{\infty }\right)$]^2^ vs. E (eV), and the Urbach energy (E_U_) was calculated by plotting ln($f\left(R_{\infty }\right)$) vs. E (eV) in the near-absorption edge [48]. The steady-state photoluminescence (PL) spectra were acquired at r.t. with a spectrofluorimeter Edinburg Instrument FLS-980, using a 300 nm excitation source (Xe lamp) for steady-state measurements. Time-resolved PL (TRPL) experiments were performed using time-correlated single-photon counting (TCSPC), measuring the emitted photons at 641 nm with a 371.8 nm pulsed diode laser as excitation source.

Attenuated total reflectance (ATR) IR spectra were obtained with a Bruker Vertex 70 spectrophotometer equipped with the Harrick MVP2 ATR cell, with resolution of 4 cm^−1^. FT-Raman spectra were obtained with the same instrument equipped with the RAMII accessory via excitation with a 1064 nm laser, with a resolution of 4 cm^−1^.

X-ray photoelectron spectroscopy (XPS) measurements were performed on a Perkin-Elmer Φ 5600ci spectrometer. The samples were analyzed using a non-monochromatic Al Kα radiation (1486.6 eV) in the 10^−6^ Pa pressure range. The analyzed sample area was around 0.5 mm^2^. In addition to the wide range survey spectrum, single spectra were recorded for Zn2p, ZnLMM, O1s, Ti2p and O1s regions. All the binding energy (BE) values are referred to the Fermi level. The correct calibration of the BE scale was verified by checking the position of both the Au4f_7/2_ and Cu2p_3/2_ bands (from pure metal targets), falling at 84.0 eV and 932.6 eV, respectively. The raw spectra, after a Shirley-type background subtraction, were fitted using a non-linear least-square fitting program adopting Gaussian–Lorentzian peak shapes for all the peaks (XPSPEAK41 freeware software, version 4.1 (accessed on 25 December 2022). Due to the presence of surface charging, samples presented a shift in the bands toward higher Bes (of around 2 eV): the charging effect was corrected by using an internal reference (Zn2p_3/2_ band centered at 1021.4 eV in ZnO compound) [49]. The uncertainty of the determined BE values was not larger than 0.2 eV. The atomic composition of the sample analyzed region (about 5-10 nm of thickness from the surface) was evaluated using sensitivity factors, as provided by Perkin-Elmer internal standard V5.4A software (accessed on 25 December 2022), after a non-linear least-square fitting process to calculate the area of the different XPS bands. A benchmark ZnO (assay >99.99%, Carlo Erba) was used as pure and crystalline reference material.

Thermo-gravimetric measurements (TG) coupled with differential thermal analysis (DTA) were carried out on NETZSCH STA 409 PC/PG instrument in air flow (20 mL∙min^−1^) in the temperature range r.t. −1000 °C (ramp: 10 °C∙min^−1^).

### 3.4. Photocatalytic Tests

The CO_2_ photoreduction tests were carried in gas-phase using a flat-type glass photoreactor, according to a previously published procedure [25]. Briefly, 10 mg of the samples were deposited onto the internal wall of the photoreactor. The reaction tests were carried out using a CO_2_/H_2_O (13.3 molar ratio) reaction mixture and in static conditions for 6 h. A 125 W medium-pressure Hg lamp (Helios Italquartz, Italy) was used as light source, with a 365 nm main emission line, a measured light intensity of 60 W∙m^−2^ and providing a heat flux as well, affording a measured reaction temperature of 70 °C. The reaction products were quantified at the end of the reaction by gas chromatography, using a 6890 HP instrument equipped with a Porapak Q packed column, a TCD detector and an automatic sampling valve. Each sample was tested twice. The blank tests were carried out using the same conditions but with a CO_2_-free gas mixture (He/H_2_O with 13.3 molar ratio).

The CO_2_ capture–photoconverting tests were performed with a CO_2_-free reaction mixture, as described for the blank test. After the first reaction, the material was left on a 10 mol. % CO_2_/He stream for 18 h in the dark before re-irradiating it with the CO_2_-free reaction mixture. This procedure was repeated three times (scheme in Figure S1).

The photocatalytic activity was reported as turnover frequency (TOF), where mol_P_ are the detected moles of reaction product, m_CAT_ the mass of the used photocatalyst and τ the reaction time:

$$\mathrm{TOF}=\frac{{\mathrm{mol}}_{P}}{m_{\mathrm{CAT}}\cdot \tau }$$

The specific photocatalytic activity (TOF*) was reported as turnover frequency normalized per specific surface area of the photocatalyst (SSA):

$${\mathrm{TOF}}^{*}=\frac{{\mathrm{mol}}_{P}}{m_{\mathrm{CAT}}\cdot \tau \cdot \mathrm{SSA}}$$

## 4. Conclusions

In this work, we synthetized ZnO materials through a wet-chemistry method, using two different annealing temperature (400 °C or 600 °C) and adding a TiO_2_ promoter in two different ways. The samples have been characterized to obtain information concerning their structural, morphological, surface and opto-electronic properties to correlate them with the synthesis conditions and, finally, to the photocatalytic activity performances. A band diagram has been also proposed, aiming to give a picture of the defect-related energetic level of the ZnO reported in this work.

The higher annealing temperature (6CSZ) results in a material with larger crystallites, O-rich surface areas, and longer excited charge carrier lifetimes, leading to a more photo-active surface. It is observed that the addition of a TiO_2_ promoter has effects which strongly depend on the synthesis method. The post-annealing addition (T4CSZ) does not alter the crystallite size and shape but leads to a surface decorated with crystalline TiO_2_ NPs and exhibits an improved charge carrier lifetime. This somewhat improved the stability against photo-corrosion compared to the unpromoted ZnO (4CSZ), but does not allow for higher activity, probably due to a surface with less affinity to CO_2_ and which, according to the band diagram, is unable to efficiently transfer the excited electron to the reagent. Conversely, adding pre-annealing TiO_2_ (4TCSZ) resulted in smaller crystallites, a more porous material and a lower TiO_2_ coverage than T4CSZ. The lower surface activity and the higher sensitivity to photo-corrosion has been attributed to a lower amount of surface hydroxyl groups and/or oxygen vacancies, which may play an important role. Finally, the ZnO photocatalytic material reported in this study, has been observed to be an effective hybrid adsorbing–photoconverting system, enabling the dilution of CO_2_ streams in the dark and its subsequent photo-conversion in CO_2_-free reaction medium.

Further studies, however, are required to gain a deeper understanding of the nature and role of surface functional groups (i.e., hydroxyl or carbonate moieties) and native point defects on the activity, CO_2_ adsorption capability and resistance against photo-corrosion of ZnO materials. Such studies could involve either other characterization techniques (i.e., electrochemical impedance spectroscopy, time-resolved absorption spectroscopy, in situ FTIR) or could investigate more synthetic variables such as different annealing temperatures, amounts and methods of the TiO_2_ promoter or a different promoter (i.e., Al_2_O_3_ or ZrO_2_).

## Figures and Tables

**Table 1:** Crystallite size determined by XRD analysis and specific surface area (SSA) determined by N_2_ physisorption of ZnO-based materials.

Sample	Crystallite Size (nm)	SSA (m^2^·g^−1^)
4CSZ	18	35
6CSZ	42	13
T4CSZ	17	33
4TCSZ	14	51

**Table 2:** XPS atomic surface composition (O, C and Zn) of ZnO materials.

Sample	O at. %	O_a_/O_b_ Ratio	C at. %	C_b_ at. %	O/Zn at. Ratio
4CSZ	41	2.3	7.5	1.7	0.79
6CSZ	44	2.0	5.3	1.4	0.89
T4CSZ	40	2.2	4.8	1.2	0.76
4TCSZ	40	2.7	4.6	1.2	0.76

**Table 3:** Tri-exponential lifetime parameters of the TR-PL decay spectra reported in Figure 5b.

Sample	τ_1_ (ns)	τ_2_ (ns)	τ_3_ (ns)
4CSZ	0.2	5.2	93.7
6CSZ	5.1	38.2	325.9
T4CSZ	2.9	27.3	264.7

**Table 4:** Labels for the synthetized ZnO materials.

Sample	TiO_2_ Promotion	Annealing Temperature and Time (°C; h)
CSZ	/	/
4CSZ	/	400; 4
6CSZ	/	600; 4
T4CSZ	On annealed ZnO (4CSZ) (post-annealing addition)	400; 1
4TCSZ	On unannealed Zn hydroxy carbonate (CSZ) (pre-annealing addition)	400; 4

## Data Availability

Not applicable.

## Supplementary Materials

The following supporting information can be downloaded at: https://www.mdpi.com/article/10.3390/molecules28124798/s1. The supplementary materials contains the scheme for hybrid capture-photoconverting reaction of CO_2_ (Figure S1), the TG-DTA and XRD analyses of the unannealed zinc hydroxy carbonate (Figure S2), the Raman spectra (Figure S3), the SEM-EDX images of Ti-containing samples (Figures S4 and S5), the XPS survey spectrum and Ti2p3/2 signals (Figures S6 and S7), the deconvolution of ATR-FTIR spectra (Figure S8) and of PL spectra (Figure S10 and Table S2), the Tauc and Urbach plots of UV-vis analyses (Figure S9, Table S1) and some supplementary data for the reactivity tests (Figures S11 and S12).
