# Supplementary material for: Effect of the Synthetic Parameters over ZnO in the CO2 Photoreduction
Source: Molecules. 2023 Jun 16;28(12):4798. doi: 10.3390/molecules28124798 (PMC10301045; doi:10.3390/molecules28124798)
Supplement: Supplementary file 1 [file molecules-28-04798-s001.zip › molecules-2423660-supplementary.pdf]

Effect of the Synthetic Parameters over ZnO in the CO_2 Photoreduction

Danny Zanardo [1,2], Giulia Forghieri [1], Elena Ghedini [1], Federica Menegazzo [1], Alessia Giordana [3], Giuseppina Cerrato [3], Elti Cattaruzza [4], Alessandro Di Michele [5], Giuseppe Cruciani [6] and Michela Signoretto [1,*]

[1] CATMAT Lab, Department of Molecular Sciences and Nanosystems, Ca' Foscari University of Venice and INSTM-RU Ve, 30172 Venice, Italy; danny.zanardo@unive.it (D.Z.); giulia.forghieri@unive.it (G.F.); elena.ghedini@unive.it (E.G.); federica.menegazzo@unive.it (F.M.)
[2] Center for Sensors and Devices, Fondazione Bruno Kessler, 38123 Trento, Italy
[3] Department of Chemistry and NIS Interdept. Centre and Consortium INSTM, University of Turin, 10125 Turin, Italy; alessia.giordana@unito.it (A.G.); giuseppina.cerrato@unito.it (G.C.)
[4] Department of Molecular Sciences and Nanosystems, Ca' Foscari University of Venice, 30172 Venice, Italy; elti.cattaruzza@unive.it
[5] Department of Physics and Geology, University of Perugia, 06123 Perugia, Italy; alessandro.dimichele@unipg.it
[6] Department of Physics and Earth Science, University of Ferrara, 44122 Ferrara, Italy; cru@unife.it
* Correspondence: miky@unive.it

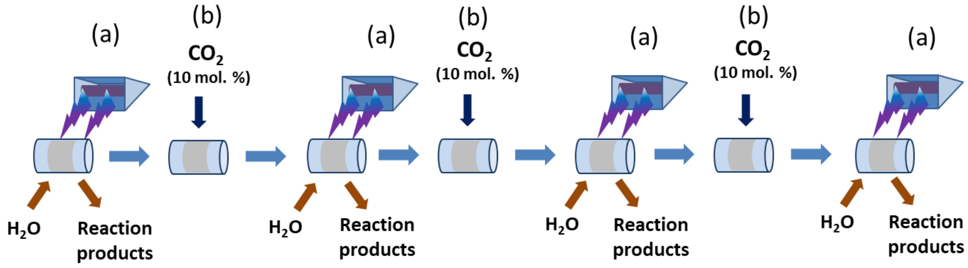

Figure S1. Hybrid CO_2 capture-photoconverting reaction scheme. (a) light-dependent photo-catalytic reaction and (b) dark CO_2 flow steps.

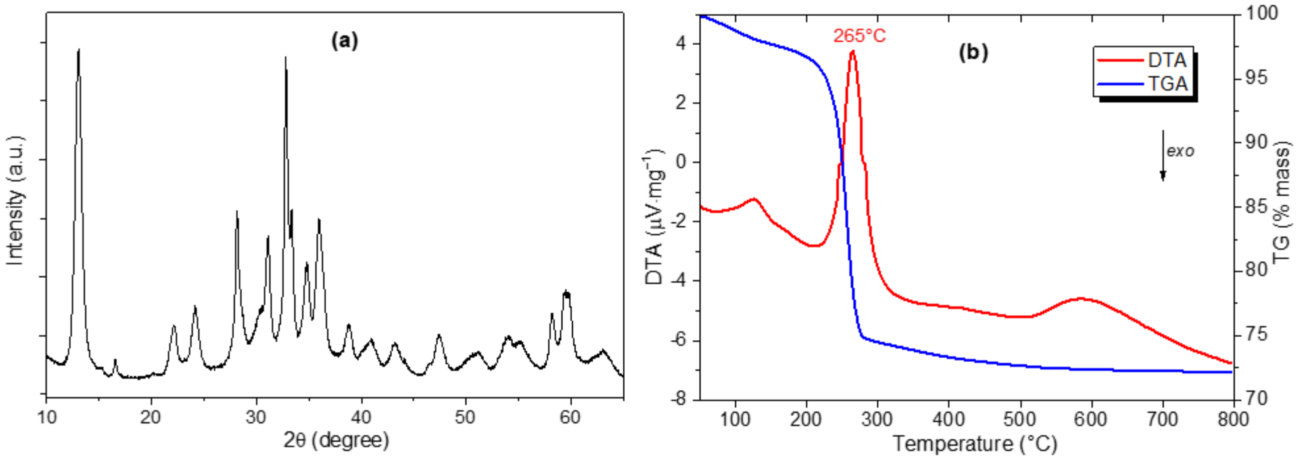

Figure S2. (a) XRD diffractogram and (b) TG-DTA of un-annealed zinc hydroxy-carbonate (CSZ).

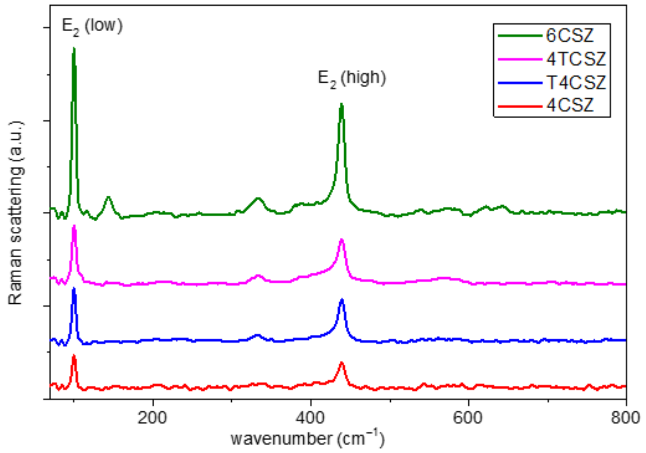

Figure S3. Raman spectra of synthetized ZnO samples.

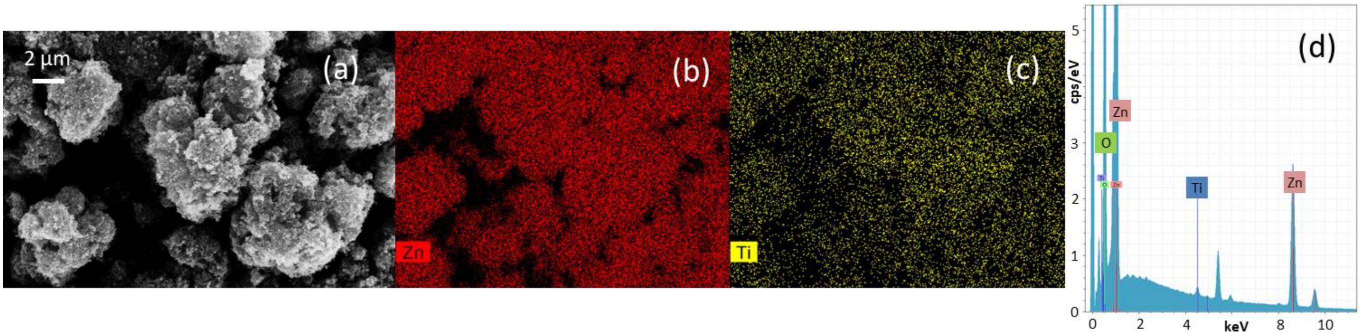

Figure S4. (a) SEM image, (b) Zn elemental mapping, (c) Ti elemental mapping and (d) EDX spectrum of T4CSZ.

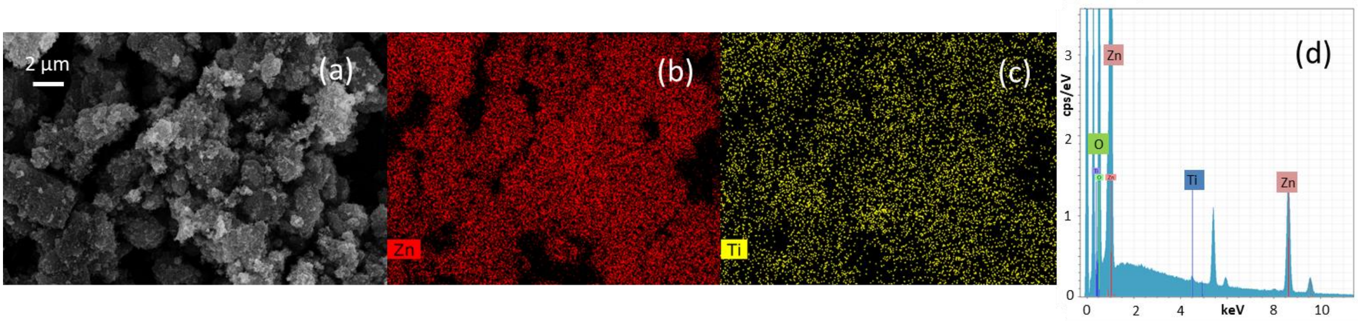

Figure S5. (a) SEM image, (b) Zn elemental mapping, (c) Ti elemental mapping and (d) EDX spectrum of 4TCSZ.

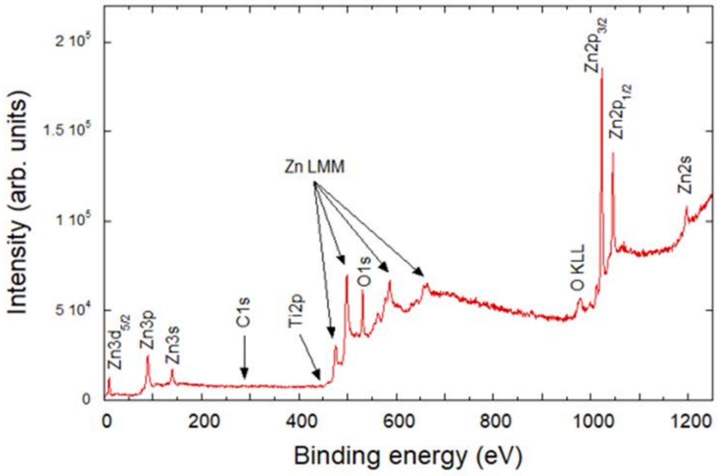

Figure S6. XPS survey spectrum of sample 4TCSZ.

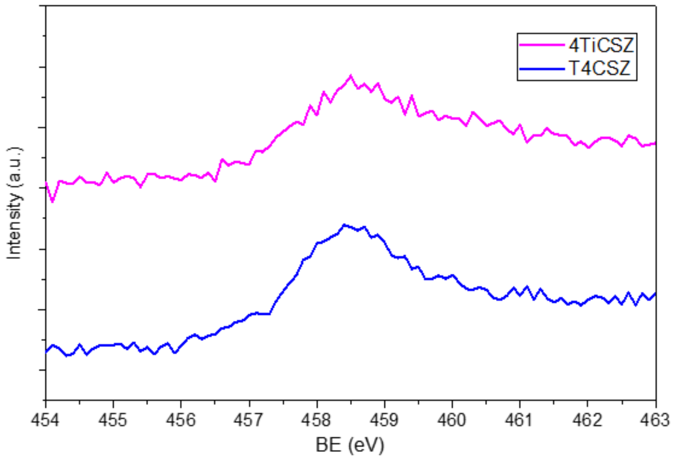

Figure S7. Ti2p$_{3/2}$ signals of T4CSZ and 4TCSZ samples.

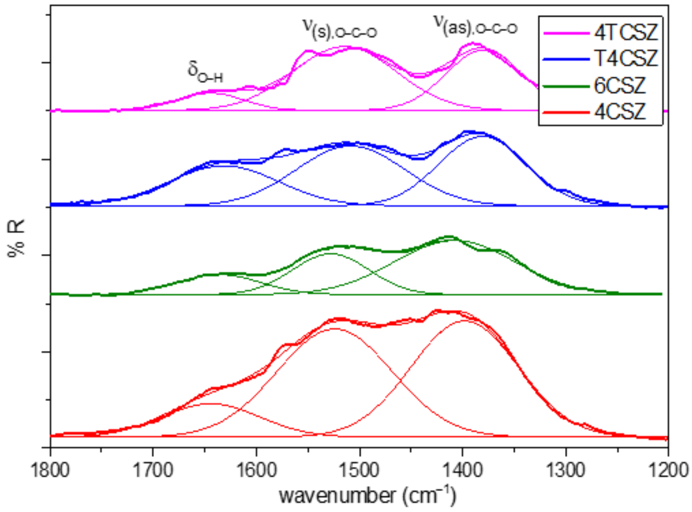

Figure S8. ATR-FTIR 1800-1200 cm^{-1} spectral region of ZnO-based samples. The non-linear fitting was performed by means of Gaussian functions.

Table S1. Band-gap (E_g) and Urbach energy (E_U) values obtained from the respective plots in Figure S8a and S8b.

**Sample**	**E_g (eV)**	**E_U (eV)**

4CSZ	3.27	0.07
6CSZ	3.28	0.06
T4CSZ	3.27	0.07
4TCSZ	3.27	0.07

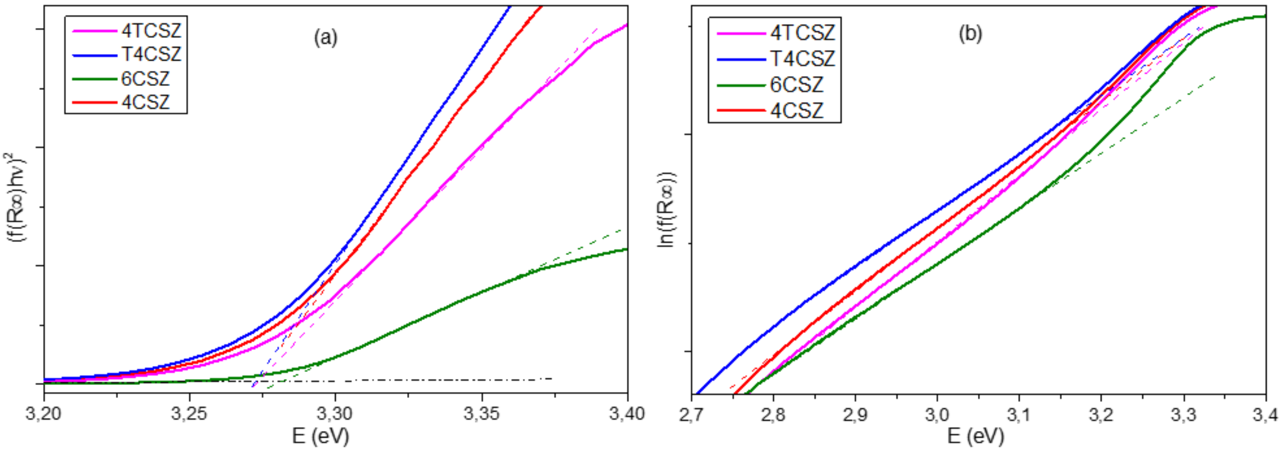

Figure S9. (a) Tauc plot and (b) Urbach plot of ZnO-based samples.

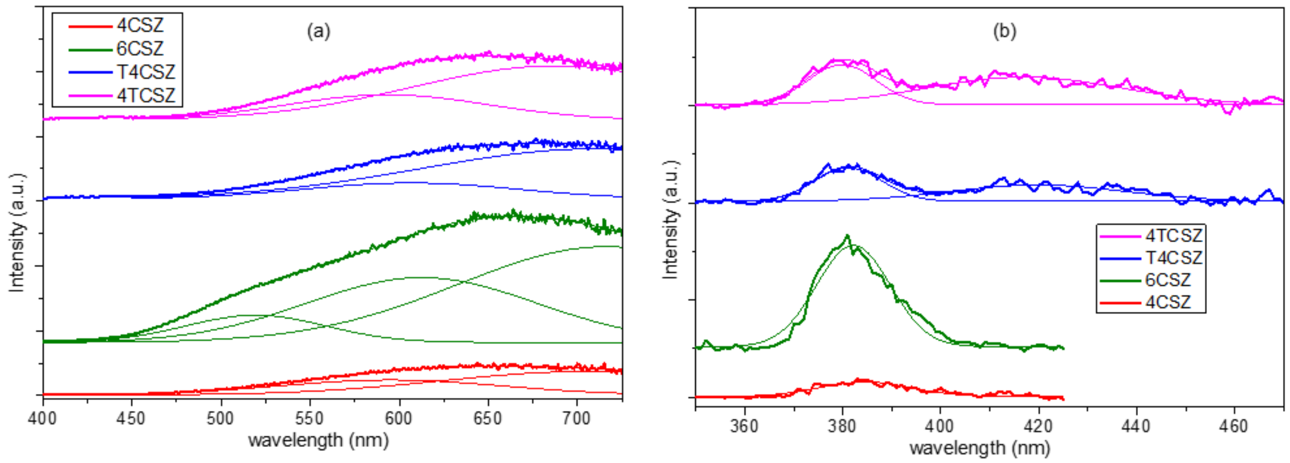

Figure S10. (a) visible range and (b) near-UV range deconvolution of PL spectra of ZnO-based samples. The non-linear fitting was performed by means of Gaussian functions.

Table S2. Areas (arbitrary units) and UV/ratio of deconvoluted UV and visible emission bands reported in Figure S10.

**Sample**	**UV/vis ratio**	**UV band** *(380 nm)*	**Violet band** *(420 nm)*	**Green band** *(520 nm)*	**Orange band** *(600 nm)*	**Red band** *(700 nm)*
4CSZ	$3.1\cdot10^{-3}$	$3.5\cdot10^{3}$	/	/	$3.7\cdot10^{5}$	$7.4\cdot10^{5}$
6CSZ	$3.8\cdot10^{-3}$	$19\cdot10^{3}$	/	$4.1\cdot10^{5}$	$16\cdot10^{5}$	$31\cdot10^{5}$
T4CSZ	$2.6\cdot10^{-3}$	$5.9\cdot10^{3}$	$7.4\cdot10^{3}$	/	$3.7\cdot10^{5}$	$19\cdot10^{5}$
4TCSZ	$3.2\cdot10^{-3}$	$7.2\cdot10^{3}$	$14\cdot10^{3}$	/	$5.5\cdot10^{5}$	$17\cdot10^{5}$

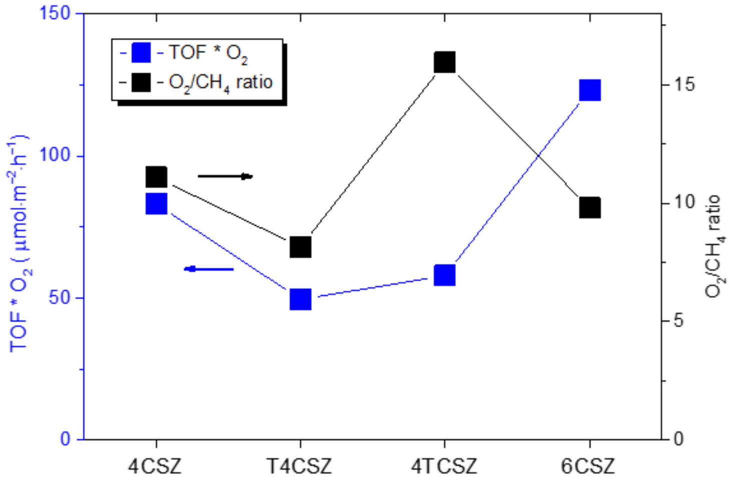

Figure S11. TOF of O_2 normalized per unit of SSA (TOF*) (blue) and O_2/CH_4 ratio of ZnO samples.

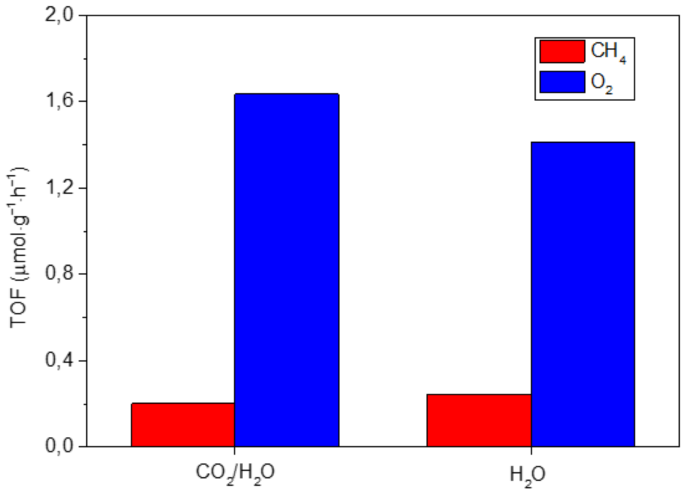

Figure S12. TOF of CH_4 and O_2 on synthetized ZnO (T4CSZ) in CO_2/H_2O and H_2O gas-phase reaction media.
